# Assessing empathy in healthcare services: a systematic review of South American healthcare workers’ and patients’ perceptions

**DOI:** 10.3389/fpsyt.2023.1249620

**Published:** 2023-11-24

**Authors:** Jeel Moya-Salazar, Eliane A Goicochea-Palomino, Jorge Porras-Guillermo, Betsy Cañari, Alexis Jaime-Quispe, Nahomi Zuñiga, María Jesús Moya-Salazar, Hans Contreras-Pulache

**Affiliations:** ^1^Faculties of Health Science, Universidad Privada del Norte, Lima, Peru; ^2^School of Biomedical Engieneering, Faculties of Engineering, Universidad Tecnológica del Perú, Lima, Peru; ^3^Digital Transformation Center, Universidad Norbert Wiener, Lima, Peru; ^4^Neuroscience Unit, Nesh Hubs, Lima, Peru

**Keywords:** empathy, healthcare workers, health services, physician-patient relations, quality of health care, South America

## Abstract

**Background:**

Empathy in healthcare service refers to the ability of healthcare workers (HCWs) to put themselves in patients’ shoes, which is necessary to ensure a good physician-patient relationship and provide quality care. Various studies have shown that empathy varies depending on the country, the instrument used, the evaluator, and the HCW’s specialty. This systematic review aims to estimate the levels of empathy among HCWs in South American countries between 2000 and 2019.

**Methods:**

We conducted searches in 15 databases (PubMed, Scopus, Web of Science, EMBASE, Scielo, PsycoInfo, ScientDirect, Latindex, and LILIACS), four preprint servers (medRxiv, bioRxiv, SportRxiv, and Preprints), and other search engines such as Dimensions (20), Google Scholar, Yahoo!, and Alicia CONCyTec (c). We followed the PRISMA guidelines, and this study was registered in PROSPERO (CRD42023454007).

**Results:**

Out of 18,532 documents identified from November 10 to 28, 2021, 10 articles were included (n = 2,487 participants, of which 1989 were patients). Among the studies focusing on self-evaluated empathy, four relied on the Jefferson Scale of Empathy for medical professionals (JSE-HP). However, assessments from patients employing Jefferson Scale of Patient’s Perceptions of Physician Empathy (JSPPPE) and Consultation and Relational Empathy (CARE) scale suggested high levels of empathy We found that both professionals and patients perceived that empathic care was provided, often at a medium or regular level. Surgery residents presented lower levels of empathy compared to obstetrics-gynecology and pediatrics physicians.

**Conclusion:**

Empathy is crucial in determining the quality of care and patient satisfaction during healthcare services provided by HCWs. Therefore, it is important to support professionals so that the various stressful situations they encounter in their work and daily life do not negatively influence the approach they provide to patients.

## 1 Introduction

Healthcare encompasses a series of formalized processes aimed at delivering services and assistance to preserve and promote well-being (1). According to the World Health Organization (WHO), quality healthcare entails providing the most appropriate diagnostic and treatment services with minimal risk and maximal patient satisfaction (2). Consequently, patient satisfaction primarily hinges on their perception and evaluation of the received care and offered services (3). Healthcare professionals (HCWs) can evaluate themselves or undergo assessments from third parties, often patients or supervisors, to gage the quality of care (4). These assessments prominently feature empathy, denoting the ability to comprehend others’ emotions (5). In healthcare, empathy signifies HCWs’ capacity to empathize with patients, understanding the emotions stirred by their health conditions (6). It serves as the cornerstone of the physician-patient relationship and a pivotal element in delivering high-quality healthcare (7).

A recent review highlighted that empathy played a causal role in the fundamental dimensions assessing healthcare quality in 58.1% of the scrutinized studies (8). This is because high levels of empathy are linked to several beneficial outcomes, including patients feeling more at ease in expressing their symptoms, better treatment adherence, reduced conflicts with HCWs, improved medical outcomes, and ultimately, heightened satisfaction (7). Furthermore, dating back to 1979, it has been well-established that human relationships promote health by preventing diseases, whereas their absence is significantly associated with increased mortality rates (9). An empathic rapport between HCWs and patients contributes to the overall well-being of both parties (9). Conversely, the absence of empathy also impacts healthcare providers, giving rise to emotional and physical work-related issues like burnout, depression, sleep disturbances, and decreased concentration (10). This chronic work-related stress burden can lead to exhaustion and burnout, further influencing empathy levels and the overall quality of healthcare (11, 12).

Latin America, particularly South America, grapples with pervasive issues of violence and a multitude of complex social and health challenges (13). This region exhibits considerable demographic diversity and operates distinct healthcare systems across its various areas (14). Consequently, the quality of care and empathy levels among HCWs can exhibit notable variations. Empirical studies have underscored these discrepancies in perceived empathy among HCWs, where, for instance, 47.1% of Mexican physicians and 83% of Chilean nurses have reported elevated levels of empathy (15, 16). Furthermore, patients and their families have contributed to assessing the empathy of healthcare professionals in Latin America, yielding estimates that span from 32 to 80% in favor of recognizing high levels of empathy (17, 18). HCWs in South America form a diverse mosaic, characterized by variations in healthcare policies and quality, both within individual countries and across the region. Morover, the assessment of empathy levels among these HCWs remains an underexplored area, highlighting the need to gain insights from existing research on this crucial subject.

Given the importance and influence of empathy on patients, HCWs, and healthcare management, we aimed to estimate the empathy among HCWs in South American countries between 2000 and 2019. Additionally, we describe differences in empathy based on work, bibliometric, and methodological characteristics, as well as the source of evaluation (patient evaluations or self-assessments by HCWs).

## 2 Methods

### 2.1 Study design, data sources, and search strategy

This review followed the guidelines of the Preferred Reporting Items for Systematic Reviews and Meta-Analysis (PRISMA) 2020 (19) and was registered in PROSPERO(CRD42023454007). Manual searches were conducted in 15 databases (PubMed, Scopus, Web of Science, Scopus, Scielo, PsycoINFO, ScientDirect, Cochrane, Latindex, and LILIACS), four preprint servers (SocArXiv, bioRxiv, and medRxiv), and other search engines such as Dimensions, Google Scholar, Yahoo!, and Alicia CONCyTec (Peruvian thesis repository) from November 28th to November 10th, 2021 (Suppl. 1).

The database search strategy was conducted using the following search equation: ((Job description OR Work schedule) AND (Healthcare workers OR Health personnel) AND (Empathy OR Consultation and Relation Empathy)). The search query was tailored for each scientific search engine, and we utilized both Spanish and Portuguese translations when searching on platforms such as Google Scholar, LILACS, Scielo, Yahoo!, and Alicia CONCyTec (Suppl. 2).

### 2.2 Inclusion and exclusion criteria

**Figure 1:**
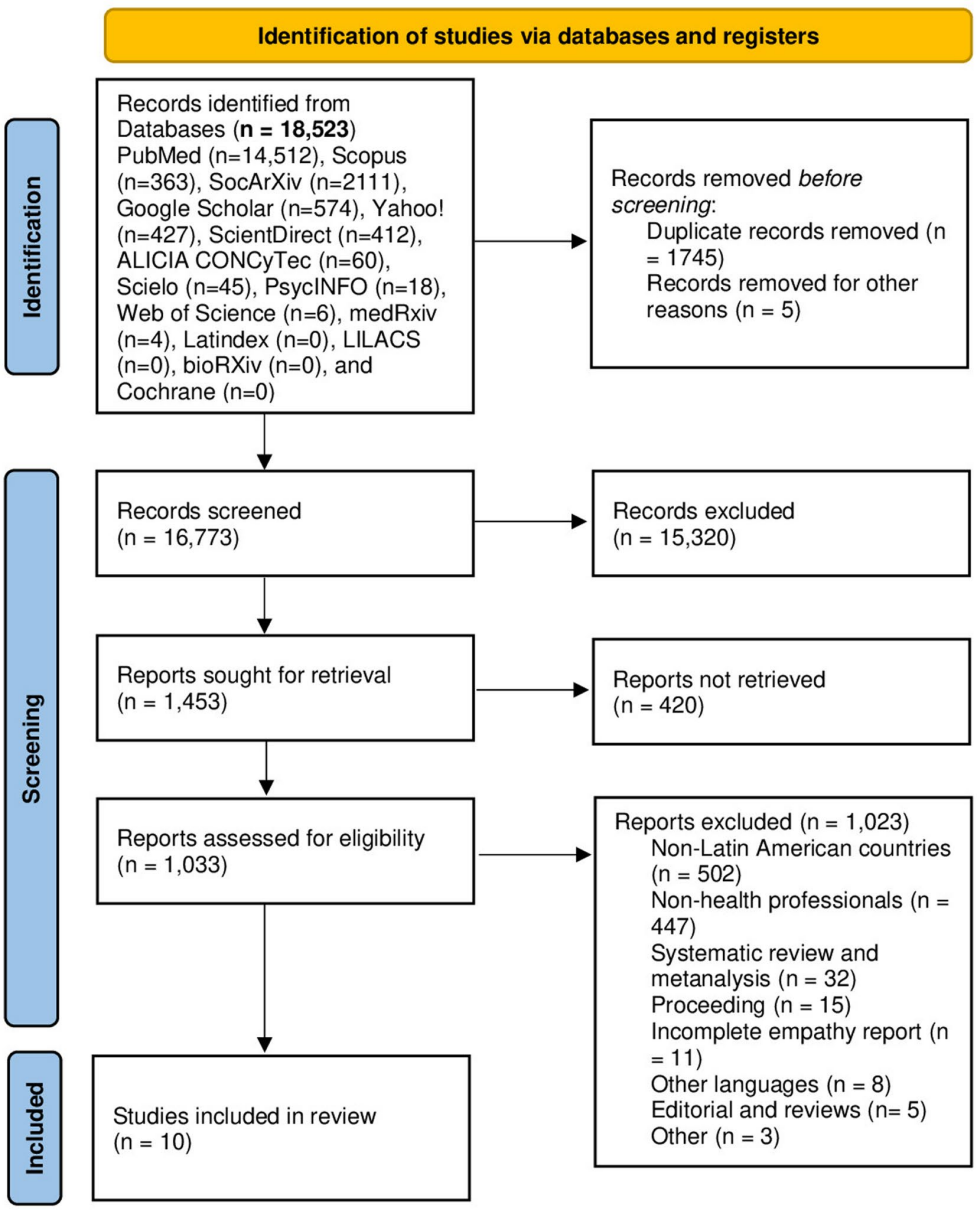
PRISMA flowchart of the study.

The included studies met the following criteria: (i) Research in HCWs on empathy, (ii) original articles, brief reports, scientific letters, and letters to the editor; (iii) articles published between 2000 and 2019; and (iv) studies involving HCWs in South America. Systematic reviews, meta-analyses, narrative reviews, scoping reviews, historical articles, reflection articles, editorials, commentaries, errata, proceedings, and case reports were excluded. Studies focusing on empathy in other populations (infants, pregnant women, older adults, individuals with physical disabilities, or conditions affecting their physical activity) or health students (medical specialty, undergraduate and graduate students) were also excluded (Figure 1).

Latin America encompasses the geographical area located to the south of the United States, comprising Mexico, the Caribbean, and South America. This diverse region is home to countries where Spanish and Portuguese are the primary languages, totaling 20 sovereign nations (e.g., Brazil) and 8 dependent territories (e.g., Puerto Rico). South America, a subregion of the Americas, stretches along the coastlines of both the Atlantic and Pacific Oceans. Comprising a total of 14 countries, it includes Argentina, Bolivia, Brazil, Chile, Colombia, Ecuador, Guyana, Paraguay, Peru, Suriname, Uruguay, and Venezuela.

### 2.3 Screening study, data extraction, and analysis

Abstracts were subjected to independent evaluation by three authors (J.M-S., J.P-G., and A.J-Q.), and any that did not meet the inclusion criteria were excluded. In accordance with the predefined protocol, these three authors also meticulously reviewed the full texts for final inclusion in the analysis. This rigorous process involved the utilization of data collection sheets and a comprehensive checklist. Disagreements were resolved through consensus at each stage of the review, and the overall agreement between reviewers was determined using the weighted Kappa correlation coefficient (20).

### 2.4 Data extraction, quality assessment, and data analysis

The process of selecting, determining eligibility, and including articles was performed manually, with three authors overseeing the flow of citations and articles throughout the review. Data was extracted from each database and exported to a data matrix using MS-Excel 2013 (Microsoft Corp., Redmond, WA, USA) and the CASPe (Critical Appraisal Skills Program) template to capture the desired information from systematic reviews (21). Bias assessment was conducted using the Cochrane risk of bias tool (Robvis 2.0), and the studies that did not contribute to the study objective (analyzing at least one variable) were considered to have a high risk of confusion and were reported. Robvis enables a comprehensive assessment of the overall confidence in the set of tests, taking into account factors such as the accuracy and relevance of the chosen studies as previously reported (22). Disagreements were resolved through consensus among the authors. Descriptive analysis of the included studies was conducted using IBM SPSS version 23.0 (Armonk, NY, USA) with frequency estimation, means, and standard deviations (SD) for categorical and continuous variables, respectively.

## 3 Results

After searching 15 databases and servers, we identified a total of 18,523 records, primarily distributed in PubMed (n = 14,512) and SocArXiv (n = 2,111). We also found 574 records in Google Scholar, 427 in Yahoo!, 412 in Sciencedirect, 363 in Scopus, 60 in ALICIA CONCyTec, 45 in Scielo, 6 in Web of Science, 18 in PsycINFO, and 4 in medRxiv. We did not obtain any results from Latindex, LILACS, bioRxiv, and Cochrane. After following the exclusion criteria and removing duplicate articles, we obtained 1,453 articles. They were then strictly evaluated based on the inclusion criteria, resulting in the inclusion of 10 articles in the systematic review (Figure 1). All included articles were conducted in South America, specifically in Brazil, Peru, Chile, and Bolivia. The total population of the 10 studies was 2,487 participants, of which 1,989 were patients.

### 3.1 Characteristics of the studies

**Figure 2:**
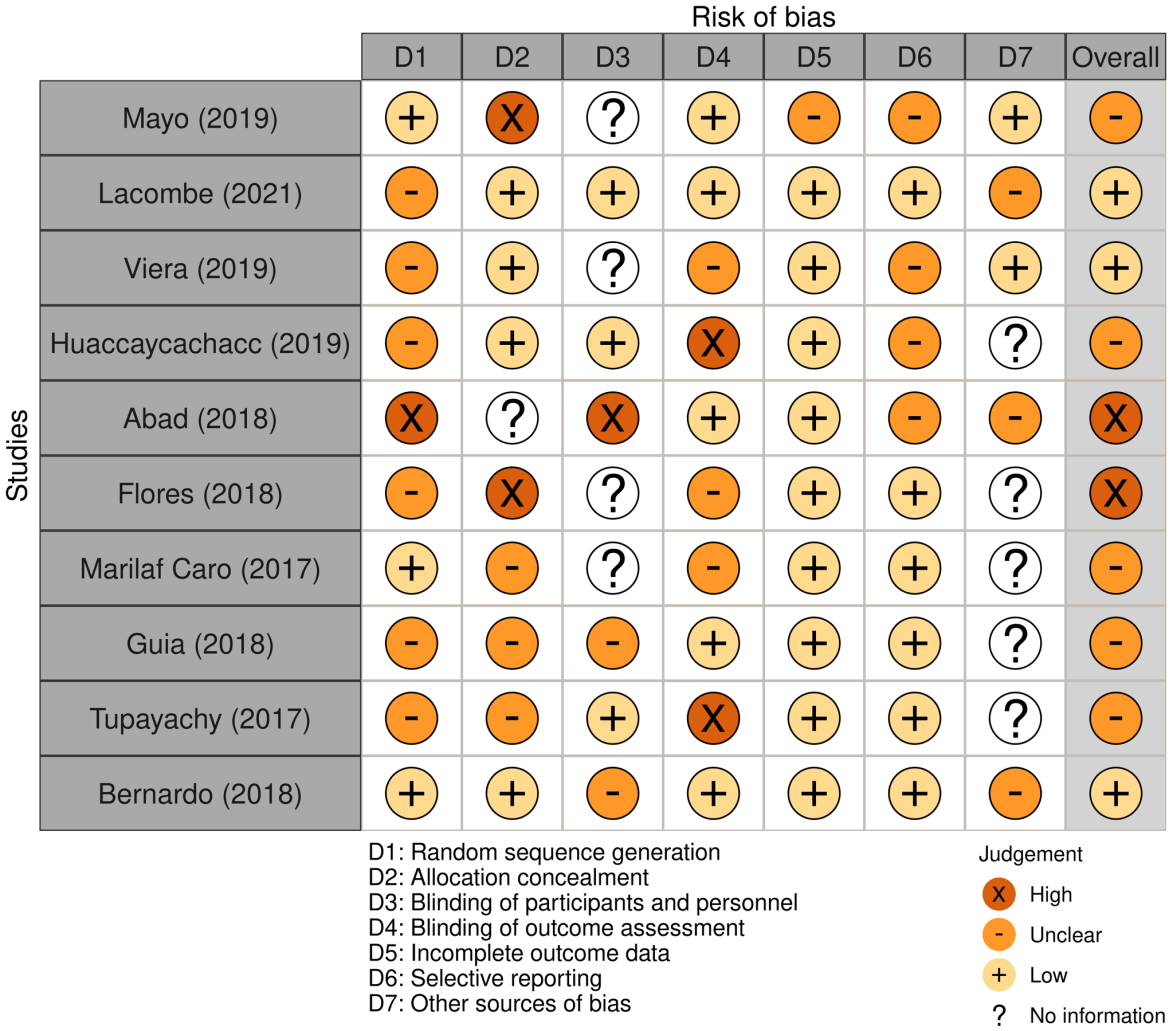
Bias analysis of included studies.

The ten studies evaluated empathy in healthcare workers from different specialties, either from the perspective of the healthcare providers themselves or from the perspective of the patients, using different approaches and instruments. Empathy during patient care tends to vary depending on the specialty and the evaluator’s perspective (see Figure 2).

Of the ten included studies, six were conducted in Peru, and two of them were cross-sectional studies that measured empathy based on self-evaluation by healthcare professionals. The study by Mayo et al. (23) included 100 resident physicians, and the study by Abad et al. (24) included 169 professionals without specifying their specialty. The rest of the studies evaluated empathy based on patient perception. Viera et al. (25) had 261 patients, Huaccaycachacc et al. (26) had 252 patients, Guia et al. (27) had 333 patients, and the observational study by Tupayachy et al. (28) included 98 oncology patients. Additionally, two studies were conducted in Brazil. The cross-sectional study by Lacombe et al. (29) included 64 interns and 50 residents. On the other hand, the observational study by Bernardo et al. (30) involved 51 physicians and 945 patients, being the only study that evaluated empathy from both perspectives. The review also included the observational study by Marilaf Caro et al. (31) conducted with 64 nursing professionals in Chile, as well as the longitudinal study by Flores et al. (32) involving 100 outpatient and hospitalized Bolivian patients (Table 1).

**Table 1:** Characteristics of the studies.

	Author (Year)	Country	Study type	*N*	Population
1	Mayo (2019)	Peru	Cross-sectional	100	Medical residents
2	Lacombe (2021)	Brazil	Cross-sectional	114	64 interns, 50 residents
3	Viera (2019)	Peru	Cross-sectional	261	Patients
4	Huaccaycachacc (2019)	Peru	Cross-sectional	252	Patients
5	Abad (2018)	Peru	Cross-sectional	169	Professionals
6	Flores (2018)	Bolivia	Longitudinal	100	Outpatients and hospitalized patients
7	Marilaf Caro (2017)	Chile	Observational	64	Nurses
8	Guia (2018)	Peru	Cross-sectional	333	33 patients per-shift = 333 cases
9	Tupayachy (2017)	Peru	Longitudinal	98	Oncology patients
10	Bernardo (2018)	Brazil	Observational	996	945 patients y 51 physicians

### 3.2 Empathy according to the evaluator

#### 3.2.1 Empathy according to healthcare providers’ self-evaluation

Out of the ten included articles, five assessed healthcare providers’ self-evaluated empathy, and only one of them also considered the perspective of the patients. The main similarity among these studies is that four of them used the Jefferson Scale of Empathy for medical professionals (JSE), which consists of 20 items in its version for healthcare providers (HP) and medical students (S). This instrument comprises three domains: compassionate care, perspective taking, and walking in patients’ shoes. Responses are rated on a 7-point Likert scale, and empathy is measured as the sum of the responses to all items. The minimum possible score is 20, and the maximum is 140 (29). The majority of the studies used the JSE-HP version. Marilaf Caro et al. (31) obtained a mean score of 126 (SD) 9, while Mayo et al. (23) reported a mean score of 112.88 (SD) 14.51 and a median score of 115. The Brazilian studies yielded similar results: Bernardo et al. (30) reported a mean score of 118.5 (SD) 14.9, and Lacombe et al. (29), using the JSE-S version, obtained a score of 118.56 (SD) 10.25. Referring to the quartiles established by Mayo et al. (23), which were 102, 115, and 124, all the studies had a medium level of empathy except for Marilaf Caro et al. (31), which had a high level of empathy. It is worth noting that Bernardo et al. (30) also used the International Reactivity Index (IRI), which was developed to measure empathy in the general population by assessing its affective and cognitive components. It consists of 28 items, and responses are rated on a 5-point Likert scale (minimum score of 28 and maximum score of 140). In contrast to the JSE, the IRI yielded a score of 58.4 ± 9.9, indicating a low level of empathy (33). On the other hand, the study by Abad et al. (24) used the Cognitive and Affective Empathy Test (TECA), which consists of 33 items. According to the participants’ gender, males showed a medium level of empathy with a percentage of 34.29%, while 26.32% of females achieved this level of empathy.

#### 3.2.2 Empathy according to patient perception

Six out of the ten included articles evaluated empathy from the perspective of the patients (one of these also assessed the perspective of the healthcare professionals). Among them, four were conducted in Peru, and two of them used the SERVQUAL tool. SERVQUAL determines service quality in healthcare facilities and medical support services. It consists of five dimensions: reliability, responsiveness, empathy, assurance, and tangibles. The study by Huaccaycachacc et al. (26) not only used questions from the modified SERVQUAL model by Parasuraman, Zeithaml, and Berry (34) but also included questions from the SERVPERF model by Cronin and Taylor (35), resulting in a total of 37 questions rated on a 5-point Likert scale. Of the total, 48.73% of the users considered the empathy provided in the hospital to be high, while 4.24% had a negative perception of empathy. Guia et al. (27) used the SERVQUAL MINSA Test for the First-Level of care, which includes 22 Expectations questions and 22 Perceptions questions, both rated on a 7-point Likert scale (36). The results showed that the surveyed professionals were empathic, and 58.0% of them were rated as having a good level of empathy.

The third Peruvian study was conducted by Viera et al. (25), who used the SERVPERF model, which has the same items and dimensions as SERVQUAL (22 items rated on a 5-point Likert scale) but excludes the part related to customer expectations. Unlike the previously mentioned studies, 45.45% of the users had a perception of “neither agree nor disagree” regarding the Empathy dimension, indicating a medium or medium level. Finally, the study by Tupayachy et al. (28) employed a self-developed questionnaire that assessed user satisfaction with healthcare services based on four dimensions: kindness, empathy and treatment, information received, and waiting times and professionalism of the staff. The majority of users expressed satisfaction with the kindness, empathy, and personal treatment provided by physicians (89.8%), nurses (84.1%), technicians (83.7%), blood bank personnel (88.5%), pathology department (73.7%), nutrition department (91.7%), and emergency services (75.6%).

The study by Flores et al. (32) used the CARE-Q (Caring Assessment Instrument) as the instrument to evaluate the service provided by healthcare professionals, based on six dimensions of behavior: accessibility, explanation and facilitation, comfort, anticipation, maintaining a trusting relationship (empathy), and monitoring and follow-up. The study found that 64% of patients expressed dissatisfaction, as they felt that the medical staff did not clarify their doubts about their illness, and perceived a lack of empathy from them.

The study by Bernardo et al. (30) used the Consultation and Relational Empathy Scale (CARE), which consists of 10 items that assess different components of empathy (affective, cognitive, and behavioral). Each item is rated on a 5-point Likert scale, where 1 is “poor” and 5 is “excellent.” The total score ranges from 10 to 50, with higher scores indicating greater empathy from healthcare professionals. In this study, the final score was found to be 42.8 ± 7.7, indicating a high level of empathy. They also used the Jefferson Scale of Patient’s Perceptions of Physician Empathy (JSPPPE), which consists of 5 items rated on a 7-point Likert scale, ranging from 1 (“completely disagree”) to 7 (“completely agree”). In this case, the score was 30.6 ± 5.6, indicating a high level of empathy as it approaches the maximum sum of the items (Table 2).

**Table 2:** Total values of empathy in the studies.

	Country	Study	Type of study	Evaluated by patients	Evaluated by the professionals	Instrument	Total values of empathy
1	Peru	Mayo (2019)	Cross-sectional		Yes	Jefferson Scale of Empathy -Health Professions (HP-version)	The total score of empathy had a mean value of 112,88 (SD) 14,51 and the median was 115.
2	Brazil	Lacombe (2021)	Cross-sectional		Yes	Jefferson Scale of Empathy - Medical students (S-version)	The final score of empathy was 118,56 ± 10,25; in which the maximum sum was 136, a number closer to the maximum value of 140 points.
3	Peru	Viera (2019)	Cross-sectional	Yes		SERVPERF model questionnaire	On average, 45.45% of the total users had a perception of neither agreeing nor disagreeing regarding the Empathy dimension.
4	Peru	Huaccaycachacc (2019)	Cross-sectional	Yes		Parasuraman, Zeithaml and Berry’s SERVQUAL	About 48,73% of users considered that empathy showed in hospital was high, and 4,24%, had a poor perception of it.
5	Peru	Abad (2018)	Cross-sectional		Yes	Cognitive and Affective Empathy Test (TECA) - 33 items	Males showed a medium level of empathy with 34,29%, while 26,32% of females obtained this level of empathy.
6	Bolivia	Flores (2019)	Longitudinal	Yes		Caring Assesment Instrument (CARE-Q)	Dissatisfaction was shown in 64% of patients, due to the fact that medical workers did not clarify their doubts regarding their illness, in addition, they perceive that these personnel do not sometimes show empathy.
7	Chile	Marilaf Caro (2017)	Observational		Yes	Jefferson Scale of Empathy -Health Professions (HP-version)	The total score of empathy had a mean value of 126 (SD) 9.
8	Peru	Guia (2018)	Cross-sectional	Yes		SERVQUAL MINSA	The surveyed professionals are empathic and 58.0% had a good level of empathy.
9	Peru	Tupayachy (2017)	Longitudinal	Yes		Self-prepared and validated questionnaire	Most of the sample were satisfied with the kindness, empathy, and personal treatment of physicians, nurses, technicians, blood bank personnel, pathology department, nutrition and emergency services.
10	Brazil	Bernardo (2018)	Observational		Yes	Physicians: Jefferson Scale of Physician Empathy (JSE) - Medical students (S-version) and the International Reactivity Index (IRI)	JSE: 118,5 ± 14,9 // IRI: 58,4 ± 9,9
11	Brazil	Bernardo (2018)	Observational	Yes		Patients: Consultation and Relational Empathy scale (CARE), and the Jefferson Scale of Patient’s Perceptions of Physician Empathy (JSPPPE).	JSPPPE: 30,6 ± 5,6 // CARE: 42,8 ± 7,7

#### 3.2.3 Empathy according to the specialty of the healthcare professional

Most of the studies evaluated empathy in the field of medicine (5/10). The Peruvian study by Tupayachy et al., (28) found that 89.8% of patients were satisfied with the physicians’ kindness, empathy, and personal attention (41,8% considered it good and 48,0% very good). In addition, Mayo et al. (23) found that 50 and 26% of medical residents had a medium and high level of empathy, respectively, with an average score of 115.5 on the JSE-HP. Lacombe et al. (29) also found a medium level of empathy among medical interns and residents, with a score of 118.56 (SD 10.25) on the JSE-S. Bernardo et al.’s study (30) found a medium level of empathy among internal medicine physicians, with a score of 120.4 (SD 11.8) on the JSE. However, when evaluating empathy based on patient perception, scores such as 31,1 ± 5,3 and 43,8 ± 7,5 were obtained on Jefferson Scale of Patient’s Perceptions of Physician Empathy (JSPPPE) and Consultation and Relational Empathy (CARE). That indicated that, according to patients, the empathy of the professionals was high. Thus, it confirmed that the self-evaluated measures of the physicians did not coincide with their patients’ perception concerning their empathy.

As for nurses’ empathy, there were different results. The study by Marilaf Caro et al. (31) recorded high empathy with a mean score of 126 (SD) 9. Tupayachy et al. (28) found that 84,1% of patients were satisfied with the kindness, empathy, and personal treatment provided by nurses (47.9% considered it good and 36.2%, very good). On the other hand, the study by Flores et al. (32) revealed that 90% of patients were dissatisfied with the service provided by nursing staff due to a lack of interest in solving their health problems and a lack of empathy; the authors concluded that nursing staff lacked empathy.

In the field of surgery, the results varied. Mayo et al. (23) found that 42.31% of surgical residents had a medium level of empathy, 38,46% had a low level, and 19,23% had a high level. In addition, they obtained a mean score of 111 on JSE-HP. On the other hand, Bernardo et al. (30) found a medium level of empathy based on self-evaluation by the professionals (JSE) with a score of 117,4 (SD) 23,2. However, when evaluating empathy based on IRI, a score of 57,3 (SD) 10.2 was obtained, which is considered low. Similarly, empathy was evaluated by patients’ perception with JSPPPE and CARE, which yielded scores of 30,0 (SD)6,0 and 42,3 (SD)7,8, respectively. Thus, according to patients, the empathy of the professionals was high (Table 3).

**Table 3:** Empathy by specialty.

Specialty	Study	Year	Results
Medicine	Mayo	2019	JSE: 115.5 // 12 residents (24%) had a low level of empathy, 25 (50%) had a medium level and 13 (26%) had a high level.
Surgery	Mayo	2019	JSE: 111 // 10 residents (38,46%) had a low level of empathy, 11 (42,31%) had a medium level and 5 (19,23%) had a high level.
Obstetrics and Gynecology	Mayo	2019	JSE: 113 // 3 residents (30%) had a low level of empathy, 6 (60%) had a medium level and 1 (10%) had a high level.
Pediatrics	Mayo	2019	JSE: 118.5 // 1 resident (7,14%) had a low level of empathy, 8 (57,14%) had a medium level and 5 (35,71%) had a high level.
Medical interns and residents	Lacombe	2021	The final score of empathy was 118,56 ± 10,25, and the máximum sum was 136, which is closer to the maximum value of 140 points.
Multi-profession	Viera	2019	On average, 45.45% of the total of users had a perception of neither agreeing nor disagreeing with respect to the Empathy dimension.
Multi-profession	Huaccaycachacc	2019	About 48,73% of the users considered the empathy showed in the hospital was high level and 4,24%, had a poor perception of it.
Multi-profession	Abad	2018	Males showed a medium level of empathy with 34,29%, while 26,32% of females had the same level of empathy.
Medicine	Flores	2019	Sixty-four percent of the patients reported being dissatisfied, due to the fact that medical personnel does not clarify their doubts regarding their illnesses; moreover, they perceive that these healthcare workers do not sometimes have empathy.
Nursing	Flores	2019	Of the total, 90% is dissatisfied with the service provided by nursing personnel, due to the fact that they do not show interest in solving their health problems, in addition to not clarifying their doubts regarding their diagnostic.
Nurses in palliative care and home care.	Marilaf Caro	2017	The total score of empathy had a mean value of 126 and a SD of 9.
Medicine, dentistry, obstetrics, nursing, nursing technicians.	Guia	2018	The surveyed professionals are empathic and 58.0% show a good level of empathy.
Medicine	Tupayachy	2017	Of the total, 89.8% felt satisfied with the kindness, empathy, and personal treatment shown during their care.
Nutrition	Tupayachy	2017	Of the total, 91.7% felt satisfied with the kindness, empathy, and personal treatment shown during their care.
Emergency	Tupayachy	2017	Of the total, 75.6% felt satisfied with the kindness, empathy, and personal treatment shown during their care.
Nursing	Tupayachy	2017	Of the total, 84.1% felt satisfied with the kindness, empathy, and personal treatment shown during their care.
Blood bank	Tupayachy	2017	Of the total, 88.5% felt satisfied with the kindness, empathy, and personal treatment shown during their care
Pathology departmen	Tupayachy	2017	Of the total, 73.7% felt satisfied with the kindness, empathy, and personal treatment shown during their care
Internal medicine	Bernardo	2018	Assessed by professionals: JSE: 120,4 ± 11,8 // IRI: 58 ± 10,9 Assessed by patients: JSPPPE: 31,1 ± 5,3 // CARE: 43,8 ± 7,5
Surgery	Bernardo	2018	Assessed by professionals: JSE: 117,4 ± 23,2 // IRI: 57,3 ± 10,2 Assessed by patients: JSPPPE: 30,0 ± 6,0 // CARE: 42,3 ± 7,8
Radiology	Bernardo	2018	Assessed by professionals: JSE: 116,4 ± 13,3 // IRI: 59,7 ± 8,8 Assessed by patients: JSPPPE: 30,1 ± 5,7 // CARE: 41,5 ± 7,8

Multi-profession: does not specify the specialty.

## 4 Discussion

This systematic review includes studies conducted in four South American countries, with 2,487 participants (1989 patients and 498 health professionals). It was found that empathy varies depending on the evaluator and the instrument used. Health workers, mostly, consider themselves with a medium level of empathy; and although some patients agree with that, others feel dissatisfied. While clinical empathy lacks a singular, universally accepted definition or standardized measurement approach (37, 38), the studies included in this analysis have made evident efforts to quantify this complex phenomenon (39, 40). In light of the inherent challenge posed by the absence of a clear and universally agreed-upon multidimensional definition of empathy, our discussion centers on a comparative examination of the findings. This approach aims to elucidate the extent of these efforts and the potential implications and influence of empathy within the healthcare context.

### 4.1 Strengths

Among the strengths of this review, we can mention that it is the first study that specifically evaluates empathy in South American healthcare professionals; other reviews do not include these countries (41) or evaluate their relationship with other variables (42). Additionally, this study has included a search in the gray literature to identify studies on empathy. It is known that these countries have limited scientific contributions, and many studies do not get published (43, 44). Therefore, this study is notable for the exhaustive search it has conducted, revealing lesser-known documents available in scientific databases. Another strength is the dual approach to estimating empathy, as it allows us to understand both patients’ perception and the self-perception of healthcare workers (HCWs). Previous studies (41, 42, 45, 46) only consider one side of the analysis, so this review broadens the perspective from both sides of the coin.

### 4.2 Overall empathy analysis

Firstly, considering the self-assessed global levels of empathy of healthcare professionals, according to the JSE for HCWs and medical students, Chilean palliative care and home care nurses in the study by Marilaf Caro et al. (31) had a high level of empathy with a mean score of 126 (SD) 9. This is due to good emotional control that allows them to cope with the daily burden of patient care. Although Brazilian medical interns and residents in Lacombe et al.’s study (29) had a medium level of empathy with a score of 118.5 (SD) 14.9, a similar relationship was observed regarding mental state. Spiritual well-being is positively associated with an empathic and patient-centered attitude.

In the Peruvian study conducted by Mayo et al. (23), a medium level of empathy was observed, with a score of 112.88 (SD) 14.51. Similar findings were reported among medical interns in two national hospitals (112.27 ± 11.85) (47) and Mexican medical students (average score of 113) (48). These results are relatively favorable when compared to a study in Greece, where HCWs in public hospitals had an average score of 102 (SD) 16.2, bordering on the lower end of the empathy scale (11). Conversely, Bernardo et al. (30) utilized two assessment instruments to gage empathy in Brazilian physicians. They obtained a medium level of empathy according to the JSE with a score of 118.5 (SD) 14.9 points, while the IRI indicated a low level of empathy with a score of 58.4 ± 9.9 (33). Similarly, Argentinean and foreign HCWs specializing in pediatric chronic diseases demonstrated a medium level of empathy, with a mean score of 82.15 (SD) 7.81 (49). Although years of professional experience do not significantly correlate with overall empathy levels, it was observed that individuals with less than 10 years of experience tend to exhibit a greater capacity for understanding another person’s perspective. Several reviews have pinpointed the factors influencing empathy and its vulnerability to fluctuations, especially in conflict environments (40). Moreover, diverse expressions and experiences of empathy exist, and its manifestation can differ among HCWs based on their roles in the healthcare system (43). Furthermore, existing evidence highlights the pivotal roles of anxiety (50) and effective patient communication (51, 52), as critical components for sustaining optimal levels of empathy in healthcare settings. Recognizing these facets is imperative for comprehending the variations in empathy levels among HCWs on a regional scale.

Chinese medical students also exhibited relatively low empathy levels (52.06 ± 10.47) (53). Notably, these studies highlight a trend where empathy levels tend to decrease as students’ progress through their medical education. This phenomenon can be attributed to the limited exposure of undergraduate students to real patients, as they have yet to directly experience the emotional demands of patient care. Although it’s been noted that study curricula in health sciences often lack clarity and sufficient support for empathy education (46), both qualitative and quantitative reviews have demonstrated that educational interventions for healthcare students can enhance empathy. These improvements subsequently lead to better-quality care during their pre-professional training or work experiences (54–56). Therefore, it is crucial to conduct regional studies in South America to explore educational practices aimed at nurturing empathy in students. The quality and human-centeredness of future healthcare depend significantly on this form of training.

Finally, the study by Abad et al. (24) used the Cognitive and Affective Empathy Test (TECA) and found that, in terms of participants’ gender, males showed a medium level of empathy with 34.29%, while 26.32% of females achieved this level of empathy. However, it was different in the high level (22.37% females vs. 11.43% males) and only females had extremely high empathy (6.58%). Previous studies indicate that females tend to be more empathic since they have skills oriented toward warm interpersonal relationships; even so, this idea is usually linked to stereotypes (45, 57, 58). These results differ from what was found in Colombian postgraduate physicians, who showed a medium level of empathy by self-evaluating themselves with the same instrument (49,6 ± 7,5), which was higher in males (52,3 ± 7,0) than in females (43.2 ± 4,4), as they had a high level of emotional understanding. Nevertheless, it is also due to the fact that most of the participants were male (59).

### 4.3 Patients’ empathy perception

In regard to the empathy level from patients’ perspective, a recent systematic review about the quality of Peruvian hospitalization care showed that it is average, due to a low empathy of healthcare workers, lack of communication about the diagnostic, treatment or possible complications of patients, in addition to the long waiting time in terms of care without justification most of the times (60). In coincidence with this review, there are two Peruvian studies, such as the one by Viera et al. (25), which used the SERVPERF questionnaire to assess the quality of service, and found that 45.45% of users treated at a polyclinic had a “neutral” perception (average level) of the staff’s empathy. This finding was also replicated in a hospital in the Municipality of Chosica in Peru, where 56% of patients perceived an average level of empathy using the SERVQUAL questionnaire (61). However, Huaccaycachacc et al. (26), who utilized both questionnaire models, found that 48.73% of users considered the empathy provided during their hospital care to be high, while 26.69% had a regular appreciation of it.

To a greater extent, Guia et al. (27), who used the SERVQUAL MINSA Test, found that 58.0 and 22.5% of the evaluated professionals (medicine, dentistry, obstetrics, nursing, and nursing technicians) had a good and excellent level of empathy, respectively. These results were correlated with the observation that professionals experience a high level of work-related stress (77.1%). On the other hand, in 2017, it was demonstrated that 56.6% of parents and guardians of pediatric patients in another Peruvian national hospital reported being satisfied with the empathy showed in the services provided. However, although the levels of empathy were deemed “acceptable,” patients did not feel entirely comfortable with the delivery of the service throughout its various stages (62). To a lesser extent, 50.6% of users felt content with the empathy in a hospital in the highlands of Peru. Moreover, this dimension was ranked second to last in terms of importance, despite it being the item that allowed physicians to better understand the patients’ health issues or the outcomes of their care (63). Tupayachy et al. (28) discovered that the majority of patients were content with the kindness, empathy, and how they were treated by medical HCWs (89.8%), nurses (84.1%), technicians (83.7%), blood bank professionals (88.5%), pathology staff (73.7%), nutritionists (91.7%), and emergency personnel (75.6%). This was attributed to the perception that the information received from these professionals was useful.

Other studies conducted in South American countries have demonstrated heterogeneous results. Bernardo et al. (30) used two instruments to evaluate empathy among Brazilian professionals: the Consultation and Relational Empathy Scale (CARE) yielded a final score of 42.8 ± 7.7, and the Jefferson Scale of Patients’ Perceptions of Physician Empathy (JSPPPE) resulted in a score of 30.6 ± 5.6. As both scores were close to the maximum sum of the items, a high level of empathy was considered. Finally, in contrast to the previously presented findings, 64% of Bolivian patients in Flores et al.’s study (32), who employed the CARE-Q, expressed dissatisfaction due to medical staff’s failure to address their concerns regarding the illness they were suffering from and their perception of a lack of empathy on certain occasions.

Given the wide range of results observed, it is essential to consider not only the high emotional involvement associated with the work of healthcare professionals but also the persisting challenges of poverty and inequity at both national and regional levels in South America, despite the economic growth and healthcare advancements achieved in the past decade. These challenges stem from inefficiencies in financing and the allocation of available budgets and resources, especially when the average of public health expenditures (GPS) in the region of the Americas is around 4% of the gross domestic product (GDP), a very low level in comparison with 8% in countries such as the USA, Canada, or the UK. It is important to highlight that most healthcare systems are fragmented, which limits access to and coverage of services. Additionally, there is a poor distribution of specialized medical professionals that does not necessarily align with the healthcare needs of different communities. Consequently, patients are often compelled to seek private services and make direct payments, placing a greater burden on individuals with limited financial resources, for whom even the smallest payment may represent a significant portion of their budget (64). All of these circumstances can become stressors that impact the empathic performance of healthcare professionals.

### 4.4 Empathy by health profession

Five out of 10 studies evaluated medical specialties in the context of empathy. In Peru, Mayo et al. (23) showed that medical residents had a medium level of empathy with a score of 115.5 according to the JSE-HP. Similar results were found in Brazilian medical interns and residents in Lacombe et al.’s study (29) with a score of 118.56 (SD) 10.25, as well as internal medicine professionals in Bernardo et al.’s study (30) who scored 120.4 (SD) 11.8. To a lesser extent, Mexican medical students interested in internal medicine specialty obtained a score of 110.3 (48). This is because internal medicine is a specialty closely related to patients, where contact starts from the beginning through structured clinical interviews, facilitating physician-patient communication. This specialty is dedicated to the comprehensive care of the sick adult, focusing on diagnosis, non-surgical treatment, and prevention of diseases affecting internal organs and systems (65). It is worth noting that, in addition to the JSE, Bernardo et al. (30) also used the IRI and obtained a low level of empathy with a score of 58 (SD) 10.9. Nonetheless, professionals working in the private sector tend to score higher in empathy, as they consciously or unconsciously modulate their behavior.

Empathy from patients’ perspective is also variable. As mentioned earlier, Bernardo et al. (30) also evaluated empathy based on patient perception using the JSPPPE and CARE, with scores of 31.1 ± 5.3 and 43.8 ± 7.5, respectively. This indicates that, according to patients, the empathy displayed by professionals was high, especially in those belonging to the private sector who have direct interaction with the patient. In Peru, Tupayachy et al. (28) found that 89.8% of patients were satisfied with the kindness, empathy, and personal treatment provided by Peruvian medical professionals (41.8% rated it as good and 48.0% as very good). Conversely, in the study by Flores et al. (32), Bolivian patients expressed dissatisfaction, with 64% stating they were unsatisfied due to a lack of clarity about their illness and occasional lack of empathy.

Nurses were the second most frequently evaluated healthcare profession. Palliative care and home care nurses in the Chilean study by Marilaf Caro et al. (31) scored high in empathy with a mean score of 126 (SD) 9 on the JSE-HP. This is particularly true for those with high emotional control, which is reflected in various aspects of their lives. These are favorable results when comparing them with Swedish nursing students who scored 113.2 (SD) 11.9 (66). However, lower scores were obtained by Iranian nurses in critical care units (87.51 (SD) 6.65), emergency departments (87.59 (SD) 4.90), and psychiatric wards (90.71 (SD) 7.12) (67). When administrating the IRI in a regional hospital in Andalusia, it was found that, despite having a low level of empathy, female nurses scored higher than males in the hospitalization unit (70.65 vs. 61.02), although the opposite was true in the intensive care unit (59.21 for males vs. 52.7 for females). Additionally, it was observed that higher levels of anxiety were associated with lower levels of empathy. This is often the case in intensive care units or emergency departments due to workload and the challenging nature of the work (68).

Two studies in Andean countries have shown differences in the empathy of nurses. First, Tupayachy et al. (28) found that 84.1% of patients were satisfied with the kindness, empathy, and personal care provided by Peruvian nurses (47.9% rated it as good and 36.2% as very good) because they found the information provided by them useful. In contrast, Flores et al. (32) found that 90% of patients were unsatisfied with the service provided by Bolivian nurses due to a lack of adequate communication mechanisms resulting from an unfavorable organizational climate. In other words, the dynamics between different professionals, in this case nurses and physicians, were deficient. Therefore, the lack of a conducive environment negatively affects job performance, thus affecting the provision of empathic healthcare services.

In surgery, Mayo et al. (23) observed a medium level of empathy among surgical residents, with a combined score of 111 on the JES-HP. Among the residents, 42.31% exhibited a medium level of empathy, 38.46% had a low level, and 19.23% demonstrated a high level of empathy. Notably, this specialty yielded the lowest empathy score among those examined in this study, in contrast to Obstetrics-Gynecology (113 points) and Pediatrics (118.5 points). The disparity can be attributed to the nature of patient-oriented specialties like pediatrics, where direct patient interaction commences from the outset. In contrast, technology-focused specialties such as surgery necessitate diagnostic procedures before patient engagement. This trend was further supported by findings in medical interns from two Peruvian hospitals; those aspiring to patient-oriented specialties exhibited higher average empathy scores compared to their counterparts pursuing technology-oriented specialties (112.09 ± 12.420 vs. 111.84 ± 10.913, respectively) (47). Even Mexican students aspiring to specialize in surgery reported even lower empathy scores (108.8) (48).

Regarding other specialties, 91.7% of patients in Tupayachy et al.’s study (28) indicated that they were satisfied with the kindness, empathy, and personal care provided by nutrition professionals; 88.5% had the same level of satisfaction with blood bank personnel, 83.7% with technicians, 73.7% with those in the pathology department, and 75.6% with emergency department personnel. Professionals in the emergency department were also evaluated in three other Peruvian hospitals, with patients indicating that 65.0 and 20% had a medium and high level of empathy, respectively (69). These differences between careers often arise due to the differences in the individuals they are associated with. Professions that are closely related to patients tend to have higher levels of empathy, especially in those with more interventions or longer patient interactions. However, it is important to consider that emergency areas often generate higher stress levels among personnel due to workload and the challenging nature of the work (23, 68). On the other hand, Bernardo et al. (30) obtained a medium level of self-assessed empathy in radiology professionals (JSE) with a score of 116.4 (SD) 13.3. However, when empathy was evaluated using the IRI, a low score of 59.7 (SD) 8.8 was obtained, indicating low empathy levels. Empathy was also assessed based on patient perception using the JSPPPE and CARE, with scores of 30.1 (SD) 5.7 and 41.5 (SD) 7.8, respectively, indicating high empathy levels in these professionals according to patients.

### 4.5 Limitations

Firstly, some studies evaluated empathy as a dimension of healthcare quality or patient satisfaction with healthcare services (25–28, 32, 60). Additionally, the relationship between empathy and other variables such as Burnout Syndrome was not considered. Burnout affects between 4.1 and 61.0% of healthcare professionals in South America (70) and is negatively associated with empathy (71). In other words, empathy decreases as burnout increases (11).

On the other hand, the studies included in this review employ various instruments to assess empathy, such as the JSE, IRI, CARE, and others. While these instruments offer diverse perspectives on empathy, their heterogeneity can make cross-study comparisons challenging. Future research should consider standardizing the assessment tools to ensure consistency and facilitate a more comprehensive analysis of empathy levels across South American countries.

The empathy during the COVID-19 pandemic was not evaluated, which generated a global crisis that negatively affected healthcare professionals, who had a higher prevalence of mental disorders such as anxiety and depression (60, 72, 73). It also led to a lack of communication between professionals and patients due to workload, resulting in deficient empathy-centered care (74). Finally, it’s possible that there are undiscovered studies conducted across various regions that incorporate samples from the South American population. This factor has the potential to impact the study’s findings and conclusions.

### 4.6 Future directions

This review provides valuable insights into the evaluation of empathy among HCWs in South American countries. However, there are several notable gaps and implications for future research and potential interventions that warrant discussion. Our results highlight a significant focus on HCWs self-assessed empathy, with only one study considering the perspective of patients. Future research should prioritize a more balanced approach by incorporating patient feedback to gain a comprehensive understanding of empathy levels. This would allow for a more holistic evaluation and provide valuable insights into the alignment or disparities between healthcare providers’ self-assessment and patient perceptions.

The review underscores the variability in empathy levels across different healthcare specialties, with some studies reporting higher empathy among certain groups of professionals. Further research should delve into the factors contributing to these variations and explore potential interventions or training programs tailored to specific specialties. This could help enhance empathy levels uniformly across healthcare disciplines. Given the importance of empathy in healthcare, future studies should focus on designing and testing intervention strategies to improve empathy among healthcare providers. These interventions could be targeted at both training programs for students and ongoing professional development for practicing professionals.

On the other hand, the included studies evaluating patient perceptions of empathy often report high levels of satisfaction, despite variations in healthcare professionals’ self-assessment (75). Future research should investigate the factors contributing to this discrepancy and whether patients’ perceptions align with objective measures of empathy. Additionally, exploring the impact of perceived empathy on patient outcomes and overall satisfaction could be a valuable avenue for research. Finally, the review highlights regional disparities in empathy levels, particularly in the context of patient perception (45). Further research should investigate the underlying factors contributing to these disparities, such as cultural influences, healthcare system characteristics, and socioeconomic factors. Understanding these nuances can guide region-specific interventions to address empathy gaps.

## 5 Conclusion

Empathy in healthcare delivery exhibits variations influenced by diverse factors, including the evaluator, healthcare specialty, and the choice of assessment tool. Notably, both healthcare professionals and patients perceive the presence of empathic care in Latin America. Nevertheless, these studies exhibit certain limitations that necessitate attention in future research endeavors. It is worth noting that not all South American nations have been extensively covered in available research, and the presence of gray literature and quality of investigations poses challenges for comprehensive systematic analyses. Addressing these issues will be pivotal in advancing our understanding of empathy in healthcare across the region.

Considering the influence of empathy on a good professional-patient relationship, it is important to provide psychological support to professionals so that workload and different situations in their daily lives do not negatively affect the care they provide to patients. Further studies should include variables related to the COVID-19 pandemic, as it can have a pivotal effect on healthcare quality.

## Author contributions

JM-S, NZ, and HC-P conceived the review topic. JM-S, NZ, JP-G, EG-P, and AJ-Q drafted the protocol, performed literature search and conducted data extraction and analysis. HC-P and MM-S contributed clinical expertise. EG-P, BC, MM-S, and JP-G contributed to manuscript authorship and editing. All authors contributed to the article and approved the submitted version.
