# Supplementary material for: Assessing empathy in healthcare services: a systematic review of South American healthcare workers’ and patients’ perceptions
Source: Front Psychiatry. 2023 Nov 24;14:1249620. doi: 10.3389/fpsyt.2023.1249620 (PMC10704173; doi:10.3389/fpsyt.2023.1249620)
Supplement: Supplementary file 1 [file Table_1.DOCX]

**Suppl 1.** Search equations used in the study

| Language | Search query |
| --- | --- |
| English | ((Job description OR Work schedule) AND (Healthcare workers OR Health personnel) AND (Empathy OR Consultation and Relation Empathy)). |
| Spanish | ((Trabajo OR Descripción del puesto OR Horario de trabajo) AND (Trabajadores de la salud OR Personal de salud) AND (Empatía OR Consulta y Relación de Empatía)). |
| Portuguese | ((Trabalho OR Descrição do trabalho OR Horário de trabalho) AND (Trabalhadores de saúde OR Pessoal de saúde) AND (Empatia OR Empatia de consulta e relacionamento)). |
