# Supplementary figures and images for: Assessing empathy in healthcare services: a systematic review of South American healthcare workers’ and patients’ perceptions
Source: Front Psychiatry. 2023 Nov 24;14:1249620. doi: 10.3389/fpsyt.2023.1249620 (PMC10704173; doi:10.3389/fpsyt.2023.1249620)

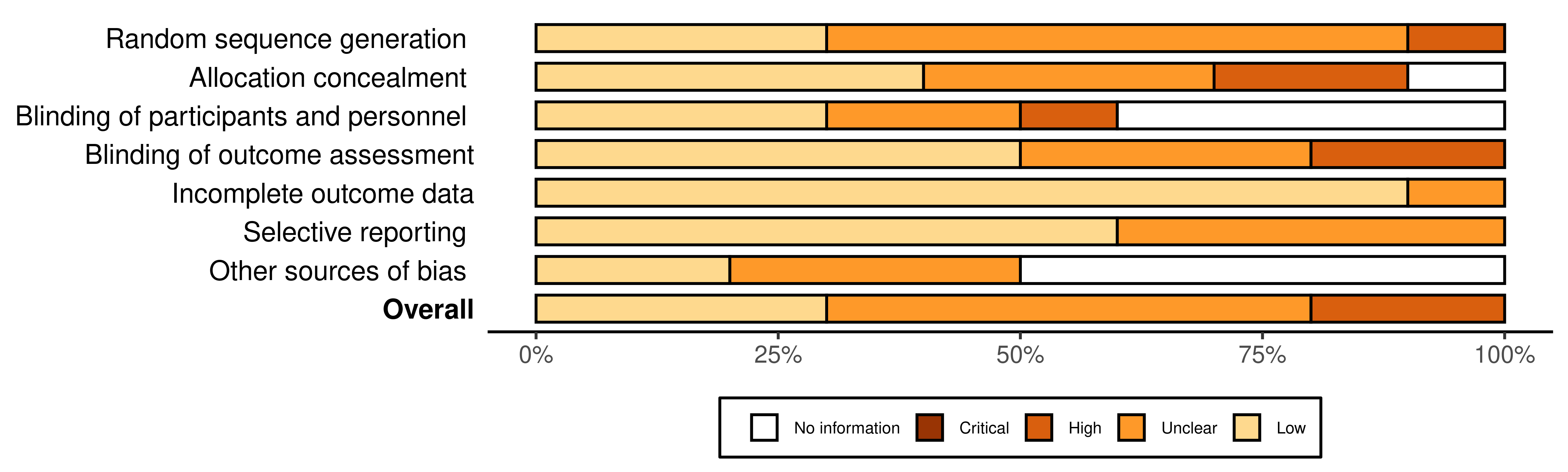

Supplement: Supplementary file 2 [file Image_1.TIFF]

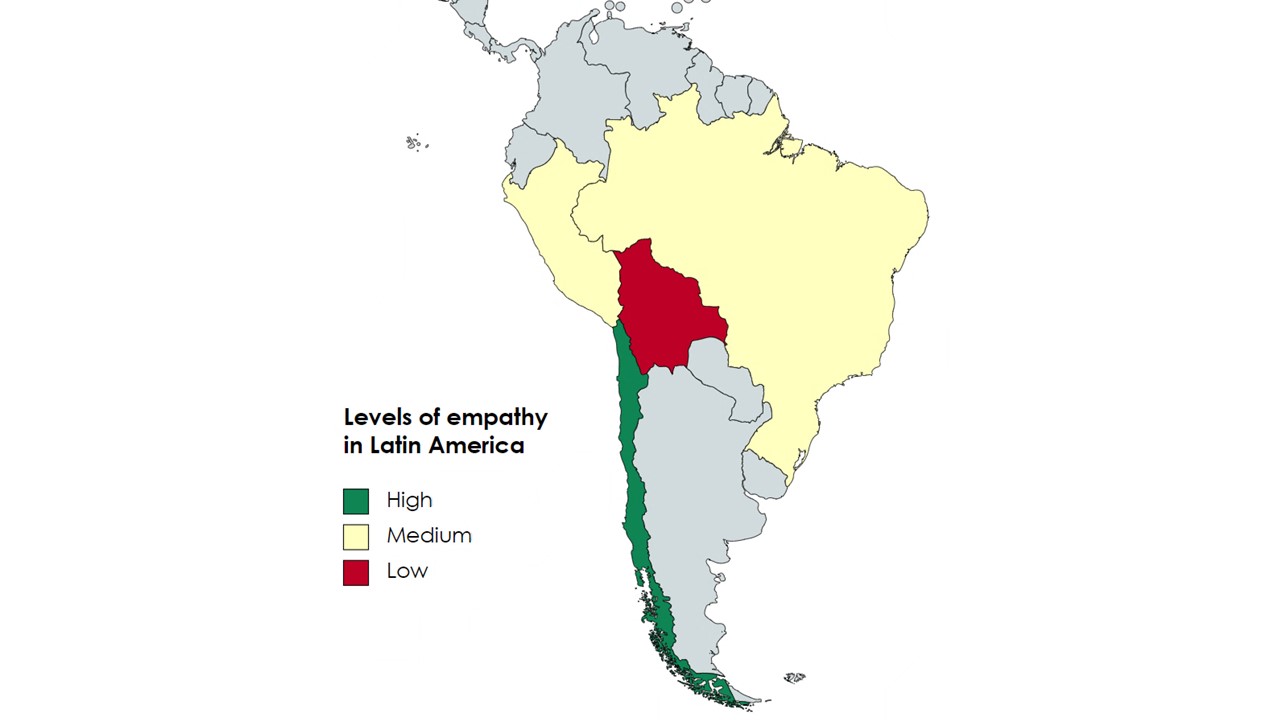

Supplement: Supplementary file 3 [file Image_2.JPEG]
